# Assessing community antibiotic usage and adherence as per standard treatment guidelines: A potential area to enhance awareness at community pharmacy settings

**DOI:** 10.1016/j.rcsop.2024.100552

**Published:** 2024-12-12

**Authors:** Abdullah Al Masud, Ramesh Lahiru Walpola, Malabika Sarker, Alamgir Kabir, Muhammad Asaduzzaman, Md Saiful Islam, Ayesha Tasnim Mostafa, Zubair Akhtar, Holly Seale

**Affiliations:** a School of Population Health, Faculty of Medicine and Health, University of New South Wales, Sydney, Australia; b School of Health Sciences, Faculty of Medicine and Health, University of New South Wales, Sydney, Australia; c Brown School of Public Health, Brown University, USA; d Heidelberg Institute of Global Health, Heidelberg University, Germany; e The George Institute for Global Health, University of New South Wales, Sydney, Australia; f Department of Community Medicine and Global Health, Institute of Health and Society, Faculty of Medicine, University of Oslo, Oslo, Norway; g BRAC James P. Grant School of Public Health, BRAC University, Dhaka, Bangladesh; h The Kirby Institute, Faculty of Medicine and Health, University of New South Wales, Sydney, Australia

**Keywords:** Antibiotic adherence, Standard treatment guidelines, Antimicrobial resistance, Bangladesh, Community pharmacies, Antibiotic nonadherence

## Abstract

**Background:**

Antibiotic nonadherence significantly contributes to poor treatment outcomes and antimicrobial resistance. In Southeast Asia, including Bangladesh, community pharmacies are crucial in primary healthcare, and are key sources of over-the-counter antibiotics. However, understanding of adherence to the full course of community-dispensed antibiotics is limited. This study measured antibiotic adherence to Bangladesh government and WHO Standard Treatment Guidelines (STGs) among patients at community pharmacies and identifies associated factors.

**Methods:**

A cross-sectional survey was conducted via phone among 358 respondents from four urban and rural areas of Bangladesh who participated in a previous antibiotic purchasing behavior survey. Descriptive analysis identified antibiotic use patterns, and adherence to the full course of antibiotics was assessed against STGs recommendations. Poisson regression model was used to explore correlations between patients' demographic characteristics, knowledge of antibiotic dosage, dosage regimen, and type of health-symptoms and adherence to the full course of antibiotics.

**Results:**

Adherence to antibiotic dosage per STGs was 40.5 %. Patients consulting a registered medical practitioner were significantly more likely to adhere (Adj-PR: 3.81, 95 % CI: 2.82–5.14) compared to those who did not. Males were 32.0 % less likely to adhere than females (Adj-PR: 0.68, 95 % CI: 0.54–0.86). Rural residents demonstrated 37.0 % lower adherence compared to urban (Adj- PR: 0.63, 95 % CI: 0.45–0.87). Respondents who recalled the antibiotic dosage had a higher likelihood of adherence (Adj-PR: 2.04, 95 % CI: 1.06–3.93). Patients on 12-hourly regimens had higher adherence (Adj-PR: 1.55, 95 % CI: 1.03–2.33) than 6-hourly regimens. Patients with uncomplicated skin-infections had higher adherence (Adj-PR: 1.72, 95 % CI: 1.22–2.47), while other symptoms showed no significant association.

**Conclusion:**

Targeted interventions in diverse healthcare settings are essential, including user-centric research and enhancing patient knowledge and involvement. Strengthening patient-physician relationships and involving community pharmacies in antimicrobial stewardship programs can improve antibiotic dispensing and counselling practices among drug-sellers.

## 1 Introduction

The misuse and overuse of antibiotics, coupled with patient nonadherence to antibiotics, significantly contribute to antimicrobial resistance (AMR),^1^ which is three to four times more prevalent in low- and middle-income countries (LMICs) compared to developed countries due to weaker regulatory frameworks.^2^^,^^3^ Besides, nonadherence to antibiotics significantly affects treatment outcomes, resulting in outcomes that are three times less effective and contributing to increased illness, higher mortality rates, and greater healthcare burdens and costs.^4^ This also diminishes the future utility of antibiotics and complicates diagnosis.^5^ A meta-analysis across four continents found that nearly half of respondents stopped their antibiotic course prematurely upon feeling better, highlighting a significant gap in efforts to ensure antibiotic adherence, resulting in AMR.^6^ However, antibiotic nonadherence rates varying widely, from 15 % to 93 %, with an average around 50 % globally.^7^^,^^8^

In many LMICs, community pharmacies play a crucial role as accessible, frequently used points of primary healthcare, offering extended hours and a wide range of medicines.^9^ However, these pharmacies often dispense antibiotics without a prescription, contributing significantly to antibiotic misuse.10., 11., 12., 13. While World Health Organization (WHO) highlights key challenges in addressing antimicrobial resistance: improving healthcare access, reducing unnecessary antibiotic use, ensuring treatment completion, and discouraging medication sharing,^14^ Southeast Asia, including Bangladesh, faces an elevated AMR risk due to suboptimal healthcare standards and the improper use of antibiotics.^15^^,^^16^ In Bangladesh, community pharmacies are predominantly privately owned, with limited dispensing areas, storage, and space for patient interactions, and approximately half operate without a license.^17^ Staff qualifications vary significantly; while some pharmacies employ trained pharmacists, many are staffed by individuals with minimal or no formal training, falling short of the ideal standard of pharmacist-led care .^17^^,^^18^ In many low-income urban and rural areas of Bangladesh, community pharmacy drug-sellers, often referred to as ‘village doctors,’ play a vital role as informal providers in the healthcare landscape.^19^ In rural areas, accessing healthcare services is challenging due to the unavailability of trained medical professionals, limited communication infrastructure, and the general unaffordability for rural residents. As a result, people in rural areas often go directly to the pharmacies as their first option for obtaining antibiotics.^20^ People rely on pharmacies for healthcare because of their flexible hours, short wait times, convenient access, and affordable medicines.^21^ Community pharmacies, which are key sources for dispensing antibiotics over-the-counter (OTC), provide 56.6 % of antibiotics at community consumer levels without a prescription.^22^ Such involvement of OTC dispensing of antibiotics through informal healthcare providers and community pharmacies contributes to irrational antibiotic utilization, posing challenges to the healthcare system in Bangladesh.^23^ Systematic reviews from studies across South Asia, Southeast Asia, South America, and Africa reveal that approximately 80 % of antimicrobial agents are used in the community, with 62 % of antibiotics globally dispensed without a prescription, particularly for non-severe conditions. This highlights a global challenge posed by OTC antibiotic dispensing and its significant contribution to irrational antibiotic use and AMR.24., 25., 26., 27.

While healthcare providers often monitor medication adherence among hospitalized patients, there is limited understanding of antibiotic nonadherence in community settings.^28^ Studies conducted in LMICs indicate that effective medication adherence interventions should target nonadherent patients and address individual barriers to adherence.^29^^,^^30^ This study aimed to measure the prevalence of antibiotic adherence according to the Standard Treatment Guidelines (STGs) set by the Ministry of Health and Family Welfare, Government of Bangladesh,^31^ and the World Health Organization (WHO)^32^ and to identify associated factors among patients obtaining antibiotics from community pharmacies.

## 2 Methods

### 2.1 Study design and data collection

This study presents data from the ‘Antibiotic Usage Behavior Survey’ (AUB-Survey) the follow-up phase of the cross-sectional ‘Antibiotic Purchasing Behavior Survey’ (APB-Survey). The methodology of the APB-Survey has been described in a previous paper.^22^ In brief, the APB-survey was conducted face-to-face with customers aged 18 years and over who visited the sampled community pharmacies to purchase antibiotics in urban and rural areas across four administrative divisions of Bangladesh between September 2022 and February 2023 to assess their antibiotic purchasing behaviours. During this survey, consent was sought for inclusion in the follow-up AUB-Survey 14–28 days later to collect information on antibiotic use by the patients for whom the antibiotics had been purchased. The inclusion criteria for this study required participants to have completed the previous APB-survey and provided consent for participation in this follow-up survey. Both surveys are linked using a unique identifier, allowing for the incorporation of some of the variables collected during the APB-survey. The survey questions were field-tested with the same participants who had been involved in the piloting of the earlier survey. Data from these pilot participants were not included in the current survey. The research team reviewed piloting experience of data collectors and addressed challenges identified during piloting, such as variations in respondents' understanding of course completion and the difficulty of lengthy survey durations. Based on feedback from the piloting, the AUB-survey tool was revised, and the number of questions was reduced. The same researchers who conducted the APB-survey also handled the over-phone data collection for the current survey, using the Qualtrics survey platform to input the data. A factsheet, containing basic information from the earlier survey, was used instead of re-asking these questions to maintain survey brevity. Only patient age, health-symptoms, and antibiotic names were reverified. Regarding antibiotic use, the specific antibiotics recorded in the earlier survey were referenced, and participants were asked about the frequency and duration of their use, rather than directly inquiring about course completion. This information was then used to define the adherence.

### 2.2 Defining adherence

Adherence to medication is a complex, multidimensional healthcare issue, with causes linked to the patient, the treatment, and/or the healthcare provider.^33^^,^^34^ The widely accepted definition of adherence is the extent to which a person's behavior, such as taking medication, aligns with healthcare provider recommendations.^35^^,^^36^ However, nonadherence can occur in various ways, including not filling prescriptions, skipping doses, doubling missed doses, or overdosing.^34^ Both WHO and World Bank identified the misuse and overuse of antimicrobials in humans, animals, and plants as one of the major drivers of AMR and the development of drug-resistant pathogens.^37^^,^^38^ Recent research highlights the overuse of antibiotics in human and veterinary healthcare as a key driver of AMR, with misuse within communities and in underprivileged areas significantly contributing to the problem.^39^^,^^40^ In formal healthcare settings, patient adherence to medication is often guided by prescriptions that specify the required dosage, supported by medical history and investigations. In contrast, for patients obtaining antibiotics without consulting registered physicians or prescriptions, determining adherence is challenging due to the lack of clear dosage guidelines. Participants in this study also took part in an earlier survey on antibiotic purchasing behavior, which revealed that around half of antibiotics were obtained without consulting a registered physician, often through self-medication based on previous experiences or recommendations from pharmacy drug-sellers or informal providers like village doctors or paramedics.^22^ Consequently, ‘adherence’ could not be determined by comparing antibiotic use with the ‘standard’ courses prescribed by registered medical practitioners, as the non-prescription groups used antibiotics without a physician's prescription. Considering these challenges and limitations, a tailored and novel approach was employed to determine adherence, using the STGs as a benchmark to define the ideal course regimen and evaluate antibiotic usage behavior.^31^^,^^32^

In this study, “adherence” was determined by evaluating patients' reported health-symptoms and their antibiotic use, comparing the frequency and duration of intake with the STGs-recommended dosage regimen for the relevant health-symptoms and antibiotic. Therefore, the study focused on how closely patients' antibiotic use aligned with these guidelines, without assessing the appropriateness of antibiotic selection from the prescribers' perspective. “Nonadherence” was categorized into two forms: underutilization, where insufficient medication is taken in terms of frequency and duration, and overutilization, where more medication is taken than specified by the STGs dosage regimen in frequency and duration. This approach applied consistently across both prescription and non-prescription groups to ensure uniformity in analysis.

### 2.3 Data analysis

Data from the AUB-Survey were cleaned and analyzed in STATA-15. Key variables from the APB-Survey were merged using one-to-one IDs to create a new dataset. To determine antibiotic adherence, three independent variables were incorporated: ‘Standard recommended duration minimum,’ ‘Standard recommended duration maximum,’ and ‘Recommended daily doses,’ based on the STGs^31^^,^^32^ in relation to respondents' reported health-symptoms and antibiotics used. The dependent variable, ‘The status of adherence,’ was assessed by comparing it to the reported antibiotic intake, including both the duration and daily doses, as the independent variables. Patients were categorized as ‘Adherence’ if their intake matched the recommended duration and daily doses, ‘Underutilized’ if intake was below the recommended minimum, ‘Over-utilized’ if it exceeded the recommended maximum, and ‘Could not determine’ if specific antibiotic recommendations were unavailable for their symptoms. ‘Underutilized’ and ‘Over-utilized’ were then recategorized as ‘Nonadherence’. During analysis, 13 patients labeled as ‘Could not determine’ were excluded due to insufficient information or their utilized antibiotics not being listed in the STGs for the reported health-symptoms, resulting in 358 respondents. Among these 358 respondents, some were caregivers for underage patients or those unable to communicate, providing information on antibiotic intake, including daily dose and duration. Therefore, respondents and patients were not mutually exclusive.

During data analysis, continuous variables such as age, education, and household income were categorized into ordinal groups as they were not linearly associated with the outcome variable, and to make results easier to interpret and communicate. The numbers and percentages of the categorical variables were calculated and compared between adherent and nonadherent groups. Pearson's Chi-squared test was used to compare characteristics between adherent and nonadherent groups. A stacked bar diagram was used to compare categories of antibiotic use with different types of antibiotics. The prevalence ratio (PR) was used to assess the association between the outcome variable, “adherence,” and the covariates such as, patients' demographic characteristics, knowledge of antibiotic dosage, dosage regimen, and type of health symptoms. PR was used as a measure of association because PR is a better measure than odds ratio in the cross sectional study where the prevalence of outcome is common.^41^ Simple and multiple Poisson regression models were used to estimate unadjusted and adjusted PRs and their 95 % CIs. Statistical significance was set at *p* < 0.05. The adjusted PRs account for potential confounders, such as age, education, household income, and other demographic factors, isolating the effect of each individual factor on adherence while reducing the influence of these confounding variables.

### 2.4 Ethics approval

The study received approval from the University of New South Wales (UNSW) Human Research Ethics Committee (approval reference- HC220360) and the Institutional Review Board (IRB) of BRAC James P. Grant School of Public Health in Bangladesh (IRB protocol number- IRB-22 September’22–037). Written consent forms in the local language were provided during the initial phase of the study, where respondents were surveyed while purchasing antibiotics at community pharmacies. During that survey, researchers provided informed consent for participation in both the initial and follow-up phases, which took place over the phone within one month. Respondents who consented to participate and signed before starting the survey were enrolled. The survey was conducted on the Qualtrics platform, with each participant assigned a unique, non-identifiable code.

## 3 Results

### 3.1 Characteristics of the respondent

Among the 358 participants in the AUB-Survey, 65.9 % were male and 34.1 % were female (Table 1).

**Table 1:** Respondent characteristics.

Characteristics	Categories	Total	Adherence n/145 (%)	Nonadherence n/213 (%)	P-Value⁎
		n/358 (%)			
Sex	Male	236 (65.9)	104 (71.7)	132 (62.0)	0.056
	Female	122 (34.1)	41 (28.3)	81 (38.0)	
Residential location	Urban	179 (50.0)	81 (55.9)	98 (46.0)	0.067
	Rural	179 (50.0)	64 (44.1)	115 (54.0)	
Age	≤ 20	43 (12.0)	20 (13.8)	23 (10.8)	0.407
	21- 30	114 (31.8)	43 (29.7)	71 (33.3)	
	31- 40	92 (25.7)	38 (26.2)	54 (25.4)	
	41- 50	61 (17.0)	29 (20.0)	32 (15.0)	
	51 and above	48 (13.4)	15 (10.3)	33 (15.5)	
Education	No formal education	50 (14.0)	6 (4.1)	44 (20.7)	<0.001
	Primary (G-5)	39 (10.9)	10 (6.9)	29 (13.6)	
	Secondary (G-10)	120 (33.5)	41 (28.3)	79 (37.1)	
	Higher secondary (G-12)	62 (17.3)	30 (20.7)	32 (15.0)	
	Graduation and above	87 (24.3)	58 (40.0)	29 (13.6)	
Monthly household income	≤ 91 US$	52 (14.5)	12 (8.3)	40 (18.8)	0.005
	92 ‐ 181 US$	128 (35.7)	46 (31.7)	82 (38.5)	
	182 ‐ 271 US$	89 (24.9)	38 (26.2)	51 (23.9)	
	272 ‐ 362 US$	40 (11.2)	20 (13.8)	20 (9.4)	
	363 ‐ 453 US$	22 (6.2)	12 (8.3)	10 (4.7)	
	≥ 454 US$	27 (7.5)	17 (11.7)	10 (4.7)	
Role of the respondent	Themselves patient	141 (39.4)	56 (38.6)	85 (39.9)	0.807
	Caregiver of a patient	217 (60.6)	89 (61.4)	128 (60.1)	

⁎ *p*-values for chi-square test.

In the adherent group, 71.7 % were male, whereas in the nonadherent group, 62 % were male. However, the difference in adherence between males and females was not statistically significant.

Urban and rural respondents were evenly split at 50 % each, with urban residents showing a slightly higher adherence of 55.9 % compared to 44.1 % for rural residents, which also was not statistically significant. The age group with the highest representation was 21–30 years, making up 31.8 % of respondents, with an adherence of 29.7 %. Adherence was similar across other age groups. Education level had a significant impact on adherence (*p* < 0.001), with those having no formal education showing the lowest adherence of 4.1 %, while those with graduation and above had the highest at 40.0 %.

Monthly household income also showed significant differences (*p* = 0.005), with the lowest income group (<91 USD) having an adherence of 8.3 %, and the highest income group (≥454 USD) having an adherence of 11.7 %. Antibiotics were purchased by 39.4 % of respondents for themselves and by 60.6 % for household members. Adherence between patients and caregivers was similar, with no significant difference.

### 3.2 Patient information and antibiotic usage patterns

Table 2 details the characteristics of patients who used antibiotics and their usage patterns. Among patients receiving antibiotics, 27.1 % fall within the age group of ≤5 years or ≥ 60 years, categorizing them as an at-risk group due to their heightened susceptibility to diseases.^42^ Patient aged 6 to 59 years constitutes 72.9 %, considered as intermediate group. The adherence difference between these age groups was not statistically significant. Most patients (72.6 %) had a single health-symptom with 69.0 % adherence, while those with two or more health-symptoms (27.4 %) had 31.0 % adherence, with no significant difference observed. The majority (92.2 %) used a single antibiotic with 93.1 % adherence, while those using two or more antibiotics had 6.9 % adherence. No statistically significant differences were observed between the two groups.

**Table 2:** Patient information and antibiotic usage patterns.

Characteristics	Categories	Total	Adherence n/145 (%)	Nonadherence n/213 (%)	P-Value⁎
		n/358 (%)			
Patient at-risk group	≤ 5 and ≥60 years (at-risk)	97 (27.1)	33 (22.8)	64 (30.1)	0.128
	6 to 59 years (intermediate)	261 (72.9)	112 (77.3)	149 (69.9)	
No. of health-symptoms	Single symptom	260 (72.6)	100 (69.0)	160 (75.1)	0.200
	Two or more	98 (27.4)	45 (31.0)	53 (24.9)	
No. of antibiotics used	Single antibiotic	330 (92.2)	135 (93.1)	195 (91.5)	0.591
	Multiple antibiotics	28 (7.8)	10 (6.9)	18 (8.5)	
Antibiotic with prescription	Yes	153 (42.7)	109 (75.2)	44 (20.7)	<0.001
	No	205 (57.3)	36 (24.8)	169 (79.3)	
Drug seller's counselling on antibiotic use	Received	315 (88.0)	127 (87.6)	188 (88.3)	0.847
	Not received	43 (12.0)	18 (12.4)	25 (11.7)	
Respondent knew about dosage	Yes	333 (93.0)	141 (97.2)	192 (90.1)	0.010
	No	25 (7.0)	4 (2.8)	21 (9.9)	
Daily frequency of antibiotics	24 hourly	81 (22.6)	39 (26.9)	42 (19.7)	0.006
	12 hourly	182 (50.8)	78 (53.8)	104 (48.8)	
	8 hourly	53 (14.8)	21 (14.5)	32 (15.0)	
	6 hourly	42 (11.7)	7 (4.8)	35 (16.4)	
Duration of antibiotics	≤ 3 days	65 (18.2)	26 (17.9)	39 (18.3)	0.380
	≤ 5 days	73 (20.4)	36 (24.8)	37 (17.4)	
	≤ 7 days	174 (48.6)	66 (45.5)	108 (50.7)	
	≥ 10 days	46 (12.9)	17 (11.7)	29 (13.6)	

⁎ p-values for Chi-square test.

Among antibiotic users, 42.7 % received prescriptions from registered medical practitioners, who have completed their bachelor's degrees in medicine and surgery (MBBS), while 57.3 % followed advice from family members, community pharmacy drug-sellers, or informal healthcare providers (village doctors). Adherence was significantly higher among those who received antibiotics with a prescription (75.2 %) from registered medical practitioners compared to those who did not (24.8 %) (*p* < 0.001). Most respondents (88.0 %) received dosage counselling from drug-sellers, achieving 87.6 % adherence, though this did not significantly affect adherence. During the survey, most respondents, 93.0 %, reported that they knew and understood the dosage (duration and daily frequency) of antibiotics. Respondents who knew about the dosage had higher adherence compared to those who did not.

Regarding the frequency of antibiotic dosage, most patients received antibiotics every 12 h (50.8 %), followed by every 24 h (22.6 %), every 8 h (14.8 %), and every 6 h (11.7 %). Adherence was highest for the 12-h regimen and lowest for the 6-h regimen, with frequency significantly affecting adherence (*p* = 0.006). The duration of antibiotic treatment varied, with most patients treated for 7 days or less (48.6 %). The duration of treatment did not significantly impact adherence.

**Fig. 1:**
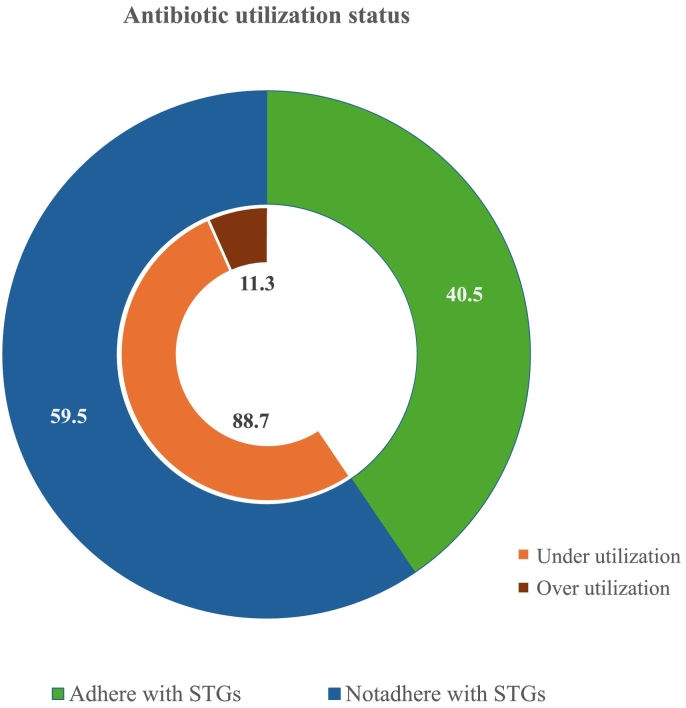
Antibiotic utilization status.

Fig. 1 illustrates the antibiotic utilization status among patients. Only 40.5 % of the total patients adhered to the dosage regimen recommended by the STGs. Among the nonadherent group, 88.7 % used antibiotics for a shorter duration than the minimum recommended, while remaining 11.3 % (24 patients) used antibiotics for a longer duration than the maximum recommended by the STGs.

### 3.3 Status of antibiotic utilization and patterns across various antibiotics

**Fig. 2:**
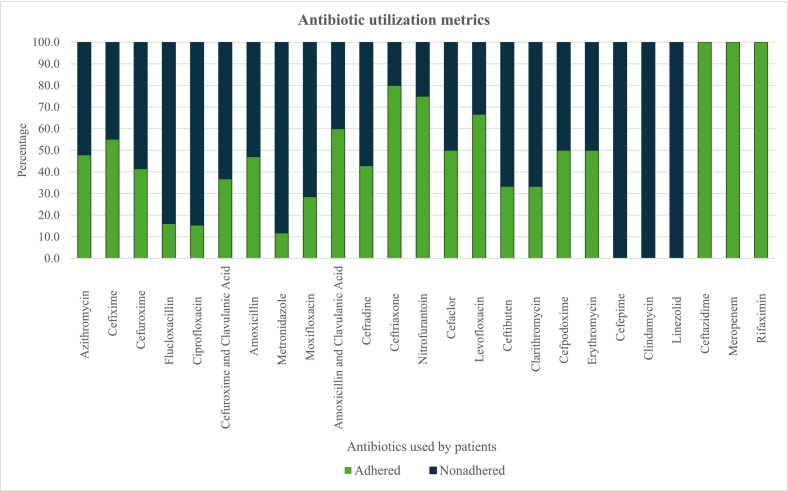
Antibiotic utilization metrics.

Fig. 2 shows adherence to antibiotic full course according to STG guidelines on antibiotic use among study patients, detailing adherence and nonadherence percentages for each antibiotic (see supplementary table 4 A and 4B for more details). Azithromycin was the most frequently used antibiotic, with 47.9 % of 71 patients adhering to the recommended dosage. Cefixime, used by 58 patients, had 55.2 % adherence.

Other antibiotics, such as Nitrofurantoin and Ceftriaxone, had higher adherence rates (75.0 % and 80.0 %, respectively) but were prescribed less frequently. Some antibiotics, like Cefepime, Linezolid and Clindamycin, showed 0.00 % adherence in their limited usage.

### 3.4 Factors associated with antibiotic adherence to the full course

Table 3 presents factors associated with adherence to the full course of antibiotics. Males were 32 % less likely to adhere the full dose of antibiotic compared to females with an adjusted PR of 0.68 (95 % CI: 0.54–0.86). Rural residents also demonstrated 37 % lower adherence compared to urban residents, with an adjusted PR of 0.63 (95 % CI, 0.45–0.87). Respondents with primary education had 48.8 % adherence, with an adjusted PR of 0.92 (95 % CI, 0.60–1.41), showing no significant difference. Those with secondary education had 39.5 % adherence with an adjusted PR of 0.63, (95 % CI, 0.44–0.91), indicating significantly lower adherence. For higher secondary education, adherence was 35.9 % (Adj. PR: 0.62, 95 % CI: 0.40–0.97), also significantly lower. Graduates or higher education had 39.0 % adherence (Adj. PR: 0.62, 95 % CI: 0.39–1.00), indicating significantly lower adherence compared to the reference group. Monthly household income between 272 and 362 US$ was associated with higher adherence, with an adjusted PR of 1.62 (95 % CI, 1.12–2.39). However, there was no trend indicated with the level of income.

**Table 3:** Factors associated with adherence to full course of antibiotics.

Predicting factors	Categories	N	Adherence n/N (%)	Crude Prevalence Ratio	Adjusted Prevalence Ratio 1, ⁎
				(95 % CI)	(95 % CI)
Sex of the respondent	female	121	58 (47.9)	Reference	Reference
	Male	237	87 (36.7)	0.76 (0.59, 0.98)	0.68 (0.54, 0.86)
Residence location	Urban	178	93 (52.3)	Reference	Reference
	Rural	180	52 (28.9)	0.55 (0.42, 0.72)	0.63 (0.45, 0.87)
Education	No formal education	47	21 (44.7)	Reference	Reference
	≤ Grade 5 (Primary)	41	20 (48.8)	1.09 (0.69, 1.70)	0.92 (0.60, 1.41)
	≤ Grade 10 (Secondary)	124	49 (39.5)	0.88 (0.60, 1.30)	0.63 (0.44, 0.91)
	H.S.C. Passed	64	23 (35.9)	0.80 (0.50, 1.27)	0.62 (0.40, 0.97)
	≥ Graduation	82	32 (39.0)	0.87 (0.57, 1.32)	0.62 (0.39, 1.00)
Monthly household income	≤ 91 US$	50	19 (38.0)	Reference	Reference
	92 ‐ 181 US$	128	49 (38.3)	1.00 (0.66, 1.52)	0.99 (0.73, 1.35)
	182‐ 271 US$	95	31 (32.6)	0.85 (0.54, 1.35)	0.83 (0.58, 1.19)
	272‐ 362 US$	38	24 (63.2)	1.66 (1.08, 2.55)	1.62 (1.10, 2.38)
	363‐ 453 US$	21	11 (52.4)	1.37 (0.80, 2.36)	1.26 (0.77, 2.05)
	≥ 454 US$	26	11 (42.3)	1.11 (0.62, 1.97)	1.12 (0.74, 1.68)
Antibiotic with prescription	Had no prescription	205	36 (17.6)	Reference	Reference
	Had a prescription	153	109 (71.2)	4.05 (2.96, 5.55)	3.81 (2.82, 5.14)
Respondent knew about dosage	No	25	4 (16.0)	Reference	Reference
	Yes	333	141 (42.3)	2.64 (1.06, 6.56)	2.04 (1.06, 3.93)
Daily frequency of antibiotics	24 hourly	81	39 (48.2)	Reference	Reference
	12 hourly	182	78 (42.9)	0.89 (0.67, 1.17)	1.55 (1.03, 2.33)
	8 hourly	53	21 (39.6)	0.82 (0.55, 1.23)	1.29 (0.78, 2.13)
	6 hourly	42	7 (16.7)	0.34 (0.16, 0.70)	0.61 (0.28, 1.33)
Duration of antibiotics	≤ 3 days	65	26 (40.0)	Reference	Reference
	≤ 5 days	73	36 (49.3)	1.23 (0.84, 1.79)	1.37 (0.95, 1.98)
	≤ 7 days	174	66 (37.9)	0.94 (0.66, 1.35)	0.76 (0.51, 1.14)
	≥ 10 days	46	17 (37.0)	0.92 (0.57, 1.49)	0.64 (0.40, 1.06)
Type of health-symptoms	Fever	32	13 (40.6)	Reference	Reference
	Upper Respiratory Tract Infection	119	37 (31.1)	0.76 (0.46, 1.25)	0.87 (0 .62, 1.22)
	Uncomplicated skin and skin structure infections	71	41 (57.8)	1.42 (0.89, 2.26)	1.72 (1.22, 2.41)
	Gastrointestinal infections	41	17 (41.5)	1.02 (0.58, 1.77)	1.19 (0.74, 1.90)
	Urinary Tract Infection	32	17 (53.1)	1.30 (0.76, 2.22)	1.60 (0.97, 2.64)
	Others	18	10 (55.6)	1.36 (0.75, 2.46)	1.21 (0.77, 1.88)
	Lower Respiratory Tract Infections	15	3 (20.0)	0.49 (0.16, 1.47)	1.20 (0.46, 3.12)
	Enteric fever	12	3 (25.0)	0.61 (0.21, 1.78)	0.96 (0.40, 2.29)
	Infection in the eyes	8	3 (37.5)	0.92 (0.34, 2.48)	1.06 (0.49, 2.27)
	Infections in the oral cavity	10	1 (10.0)	0.24 (0.03, 1.66)	0.47 (0.10, 2.18)

1 PRs were adjusted for all other variables in the table.

⁎ Supplementary Table 4C outlines various reported health symptoms and their categorization under different symptom groups.

Among the patients, who received antibiotics with due consultation of a registered medical practitioners (having a prescription), had significantly higher likelihood of adherence to the full course, with an adjusted PR of 3.81 (95 % CI: 2.82–5.14). Respondents who reported they understood the dosage of antibiotics and could recall at the time of survey had higher likelihood of adherence to the full course, with an adjusted PR of 2.04 (95 % CI: 1.06–3.93). Patients on 12-hourly antibiotic regimens had higher adherence with an adjusted PR of 1.55, 95 % CI: 1.03–2.33). Those on 6-hourly regimens had lower adherence with an adjusted PR of 0.61, 95 % CI: 0.28–1.33). Other dose-regimens showed no significant differences. Patients with antibiotic-durations of ≤5 days showed higher adherence (Adj-PR of 1.37, 95 % CI: 0.95–1.98), however the difference was not statistically significant with other durations. Adherence slightly decreased with longer antibiotic durations (≤ 7 days and ≥ 10 days), but none of the groups exhibited statistically significant differences from the reference. Individuals with uncomplicated skin and skin structure infections showed a statistically significant association with higher adherence, with an adjusted PR of 1.72 (95 % CI: 1.22–2.41). Other health-symptom categories did not demonstrate statistically significant associations with adherence.

## 4 Discussion

This study underscores that 40.5 % of patients adhered to the minimum recommended dosage of antibiotics according to STGs, which is lower than the global average of 50 %^7^^,^^8^ and another study in Bangladesh at 50 %.^43^ However, it is almost aligned with India at 36.6 %^7^ and other LMICs, such as Nigeria at 36.6 %^44^ and Ethiopia at 39.9 %.^28^ These differences may arise from variations in study design and methods for assessing adherence, or from including both hospital and community settings, whereas this study specifically focuses on community settings. In this study, individuals who obtained antibiotics with a prescription from a registered medical practitioner had significantly higher adherence compared to those without a prescription. This is consistent with other studies where participants without a prescription were twice as likely to be nonadherent.^28^^,^^45^

In this study, respondents knowledgeable about antibiotic dosage were more likely to complete the full course. Across nine studies, including one in a low- to middle-income country, antibiotic knowledge influenced self-medication and adherence to prescribed treatment. Lower knowledge, including understanding indications and antimicrobial resistance, correlated with higher self-medication. Better knowledge was associated with reduced antibiotic misuse in both high-income and low- to middle-income countries.^46^ Higher frequency antibiotic regimens of more than twice a day showed lower adherence, while shorter durations of use demonstrated higher adherence. However, these differences were not statistically significant across various regimens and durations. Another study on antibiotic non-adherence in community pharmacy settings found that lower frequency regimens were associated with reduced nonadherence risk, and longer antibiotic durations increased nonadherence risk.^45^ These findings are consistent with a systematic review of 22 studies, including four in low- and middle-income countries, which highlighted that simpler regimens with fewer doses per day and shorter treatment durations consistently improve adherence.^46^

Findings from this study indicate that socio-demographic factors, such as sex and residential location, as well as the antibiotic initiation process— including understanding of dosage, dosage frequency, and the nature of symptoms— positively influenced adherence. However, education level and income did not show a significant association with adherence. Males were found 32 % less likely to adhere to the full antibiotic course compared to females, consistent with a study conducted in Ethiopia, which showed that males were nearly twice as likely to be nonadherent.^28^ Research on gender differences in medication adherence suggests that men are generally less adherent due to factors such as fewer healthcare visits, a tendency to downplay health issues,^47^ and a perception of being healthier. Men are also more likely to discontinue antibiotics prematurely once symptoms improve,^48^ often due to a higher inclination toward risk-taking behaviours and a lower perception of illness severity.^49^ Rural residents were found to be 45 % less likely to adhere to antibiotics compared to urban residents. This contrasts with findings from a Northwest Ethiopia study, which reported higher inappropriate use among urban residents (33.1 %) than rural residents (29.2 %).^50^ This may be because rural people often receive antibiotics from informal providers like village doctors or drug-sellers with improper durations, unlike urban residents. This aligns with other studies that evaluated rural-urban differences in antibiotic prescribing.^51^ Multiple studies have identified a higher risk of antibiotic nonadherence among individuals with lower education levels.^28^^,^^46^^,^^52^ However, this study found no significant difference in adherence across education levels, aligning with research from China and Nigeria that similarly reported no association between educational level and antibiotic adherence.^44^^,^^53^ This finding contrasts with a 2019 study in Bangladesh that highlighted a significant impact of education on adherence.^8^ The inconsistency indicates that the traditional education system may failed to adequately cover proper medication or antibiotic use. Even with high education levels, a lack of understanding about antibiotics can lead to poor adherence.^54^

In hospitals, adherence can be ensured by healthcare workers and patient attendants administering antibiotics, with doctors making prescribing decisions. However, in LMICs, where nearly 80 % of antibiotics are consumed in the community,^55^ adherence relies on patients and caregivers. A study in Bangladesh suggests that nonadherence may result from insufficient communication between health providers and patients about treatment plans.^43^ To enhance adherence, healthcare providers should comprehensively explain prescription details and cultivate positive provider-patient relationships, which can encourage patients to follow their treatment regimens.^8^ Patient-centered care emphasizes considering patients' preferences, needs, and values. For antibiotics, responsible use involves prescribing the right antibiotic for the correct duration and dose, while patient-centered care includes active involvement in decision-making and providing adequate information.^46^ Studies in India and other countries show that consumers often purchase antibiotics for shorter durations than prescribed.^56^^,^^57^ Community pharmacies are the first point for healthcare seeking in many LMICs including Bangladesh^58^ and pharmacists play a critical role by dispensing antibiotics according to consumer demand to maintain business, which shifts focus away from health considerations to commercial interests. This practice by consumers and pharmacists in the community contributes to irrational antibiotic consumption.^59^ It is crucial that community pharmacies participate in antimicrobial stewardship (AMS) programs and receive adequate education and support. For instance, in Tanzania, researchers trained pharmacists to consult with customers and provide appropriate health information^60^^,^^61^ which successfully decreased inappropriate antibiotic use.^62^ Studies indicate that pharmacists should strengthen and clarify physicians' treatment instructions while identifying potential barriers to nonadherence, ensure the patient clearly understood the dose and duration of antibiotic treatment.^63^ Lack of antibiotic knowledge, irrespective of education level, is linked to nonadherence. Therefore, multilevel, culturally relevant public education is essential for combating AMR. Enhancing knowledge and engaging patients through comprehensive, community-wide interventions can significantly improve adherence and treatment acceptance.^54^

### 4.1 Limitations

One major challenge of this study was the setting. In formal healthcare systems, adherence is easier to assess due to patient records, antibiotic documentation, and investigation reports. However, in low-resource settings like Bangladesh, where many rely on informal healthcare providers without medical qualifications or authorization to prescribe antibiotics, individuals often procure antibiotics based on personal experience or without a prescription, bypassing registered medical practitioners. This survey included participants who purchased antibiotics from community pharmacies, with over half initiating antibiotics without proper consultation or prescriptions,^22^ lacking investigation reports. This absence of formal reference data made adherence assessment challenging. Due to reliance on self-reported health-symptoms and no investigation reports, it was impossible to assess causative microorganisms or infection severity, limiting the evaluation of antibiotic appropriateness. Given these context and limitations, adherence in this study was assessed by comparing self-reported health-symptoms and antibiotic use (e.g., frequency and duration) with the STGs for the relevant illness and corresponding antibiotic prescribed.^31^^,^^32^ These references provide antibiotic choice, dose, and duration based on symptom severity. For chronic conditions, the antibiotic course duration and choice may differ from non-severe conditions. Therefore, the protocols for non-severe conditions were used in this analysis, as disease severity and length of illness were not specified in the reported data. These may potentially overestimate adherence and affect representativeness. The findings are cautiously interpreted as they are based on self-reported responses.

## 5 Conclusion

This study examined socio-demographic factors, health-symptoms, sources of antibiotic administration, and dosage regimens, all of which are strongly associated with antibiotic adherence. The findings are critical for targeted interventions, particularly for patients who rely on informal healthcare providers in resource-limited settings. Additionally, the results will guide policymakers, stakeholders, and the scientific community in understanding the extent of necessary policy revisions on responsible antibiotic use both in formal and informal healthcare settings. Effective strategies to reduce nonadherence must consider social determinants of health, with targeted interventions needed in both formal and informal healthcare settings, especially in LMICs where most antibiotics are used in community settings. Adherence often depends on patients and caregivers, with poorer communication between healthcare providers and patients leading to nonadherence. Strengthening patient-physician relationships and emphasizing patient-centered care—considering preferences, needs, values, and counselling time—are crucial. Patients should actively collaborate with health professionals in managing their care, and effective communication between the two is essential for successful clinical practice. Community pharmacies must engage in AMS programs and receive proper training. Pharmacists should clarify treatment instructions and identify barriers to adherence. Strategies to enhance patient knowledge and involvement can improve adherence. Key strategies include user-centric approaches through co-designed research with stakeholders and emphasizing education and awareness. Interventions must address both consumer education and provider training on proper antibiotic use, as well as the consequences of misuse. Rigorous studies with robust methodologies and strong surveillance are needed. Urgent, multifaceted strategies are essential to tackle antibiotic misuse in LMICs.

## Declaration on the use of AI assisted tools

During the preparation of this manuscript, AI-assisted tools were used limitedly for language editing and fixing grammatical errors.

## Funding

This study is a component of the International Society of Antimicrobial Chemotherapy (ISAC)-funded project grant 2021 titled “ Practices of antibiotic consumption and dispensing in the middle and low-income people in Bangladesh: a potential contributor to the emergence of antimicrobial resistance in the community”. The funders had no role in the design, execution, or interpretation of the study.

## CRediT authorship contribution statement

**Abdullah Al Masud:** Writing – review & editing, Writing – original draft, Visualization, Validation, Software, Resources, Project administration, Methodology, Investigation, Funding acquisition, Formal analysis, Data curation, Conceptualization. **Ramesh Lahiru Walpola:** Writing – review & editing, Supervision, Resources, Methodology, Funding acquisition, Conceptualization. **Malabika Sarker:** Writing – review & editing, Supervision, Resources, Project administration, Methodology, Funding acquisition, Conceptualization. **Alamgir Kabir:** Writing – review & editing, Visualization, Validation, Supervision, Software, Methodology, Formal analysis, Data curation. **Muhammad Asaduzzaman:** Writing – review & editing, Visualization, Validation, Methodology, Funding acquisition, Conceptualization. **Md Saiful Islam:** Writing – review & editing, Methodology, Funding acquisition, Conceptualization. **Ayesha Tasnim Mostafa:** Project administration, Investigation, Formal analysis. **Zubair Akhtar:** Writing – review & editing, Visualization, Software, Formal analysis. **Holly Seale:** Writing – review & editing, Visualization, Validation, Supervision, Software, Resources, Methodology, Funding acquisition, Formal analysis, Conceptualization.

## Declaration of competing interest

The authors declare no conflicts of interest regarding the presented research. Any potential competing interests have been acknowledged and managed to maintain the study's integrity and impartiality.

## Data Availability

The datasets utilized in this study are accessible from the corresponding author upon reasonable request, in accordance with the data sharing policies of the University of New South Wales and BRAC James P. Grant School of Public Health. Supplementary files, including supporting data and summaries, are available.

## Appendix A Supplementary data

Supplementary material Image 1
