# Supplementary material for: Assessing community antibiotic usage and adherence as per standard treatment guidelines: A potential area to enhance awareness at community pharmacy settings
Source: Explor Res Clin Soc Pharm. 2024 Dec 12;17:100552. doi: 10.1016/j.rcsop.2024.100552 (PMC11731586; doi:10.1016/j.rcsop.2024.100552)
Supplement: Supplementary file 1 — Supplementary material [file mmc1.docx]

**Supplementary tables**

**Supplementary table 4A: Additional information on antibiotic utilization pattern in various antibiotics**

| **Antibiotic name**  **(Generic)** | **Total** | | **Adhered** | | **Non-adhered** | |
| --- | --- | --- | --- | --- | --- | --- |
|  | **N** | **%** | **n/N** | **%** | **n/N** | **%** |
| Azithromycin | 71 | 19.8 | 34 | 47.9 | 37 | 52.1 |
| Cefixime | 58 | 16.2 | 32 | 55.2 | 26 | 44.8 |
| Cefuroxime | 53 | 14.8 | 22 | 41.5 | 31 | 58.5 |
| Flucloxacillin | 31 | 8.7 | 5 | 16.1 | 26 | 83.9 |
| Ciprofloxacin | 26 | 7.3 | 4 | 15.4 | 22 | 84.6 |
| Cefuroxime and Clavulanic Acid | 19 | 5.3 | 7 | 36.8 | 12 | 63.2 |
| Amoxicillin | 17 | 4.8 | 8 | 47.1 | 9 | 52.9 |
| Metronidazole | 17 | 4.8 | 2 | 11.8 | 15 | 88.2 |
| Moxifloxacin | 14 | 3.9 | 4 | 28.6 | 10 | 71.4 |
| Amoxicillin and Clavulanic Acid | 10 | 2.8 | 6 | 60.0 | 4 | 40.0 |
| Cefradine | 7 | 2.0 | 3 | 42.9 | 4 | 57.1 |
| Ceftriaxone | 5 | 1.4 | 4 | 80.0 | 1 | 20.0 |
| Nitrofurantoin | 4 | 1.1 | 3 | 75.0 | 1 | 25.0 |
| Cefaclor | 4 | 1.1 | 2 | 50.0 | 2 | 50.0 |
| Levofloxacin | 3 | 0.8 | 2 | 66.7 | 1 | 33.3 |
| Ceftibuten | 3 | 0.8 | 1 | 33.3 | 2 | 66.7 |
| Clarithromycin | 3 | 0.8 | 1 | 33.3 | 2 | 66.7 |
| Cefpodoxime | 2 | 0.6 | 1 | 50.0 | 1 | 50.0 |
| Erythromycin | 2 | 0.6 | 1 | 50.0 | 1 | 50.0 |
| Cefepime | 2 | 0.6 | 0 | 0.0 | 2 | 100.0 |
| Clindamycin | 2 | 0.6 | 0 | 0.0 | 2 | 100.0 |
| Linezolid | 2 | 0.6 | 0 | 0.0 | 2 | 100.0 |
| Ceftazidime | 1 | 0.3 | 1 | 100.0 | 0 | 0.0 |
| Meropenem | 1 | 0.3 | 1 | 100.0 | 0 | 0.0 |
| Rifaximin | 1 | 0.3 | 1 | 100.0 | 0 | 0.0 |

**Supplementary table 4B: Generic antibiotics found identified in the survey under each class**

| **Antibiotic class** | **Generic name** | **ATC code** | **WHO-AWaRe classification** | **WHO essential medicine list** |
| --- | --- | --- | --- | --- |
| Macrolides | Clarithromycin | J01FA09 | Watch | Yes |
|  | Erythromycin | J01FA01 | Watch | Yes |
|  | Azithromycin | J01FA10 | Watch | Yes |
| Third-generation-cephalosporins | Cefixime | J01DD08 | Watch | Yes |
|  | Ceftriaxone | J01DD04 | Watch | Yes |
|  | Ceftibuten | J01DD14 | Watch | No |
|  | Cefpodoxime-proxetil | J01DD13 | Watch | No |
|  | Ceftazidime | J01DD02 | Watch | Yes |
| Second-generation-cephalosporins | Cefuroxime | J01DC02 | Watch | Yes |
|  | Cefaclor | J01DC04 | Watch | No |
| Penicillins | Flucloxacillin | J01CF05 | Access | Yes |
|  | Amoxicillin | J01CA04 | Access | Yes |
| Fluoroquinolones | Ciprofloxacin | J01MA02 | Watch | Yes |
|  | Levofloxacin | J01MA12 | Watch | No |
|  | Moxifloxacin | J01MA14 | Watch | No |
| Beta-lactam/beta-lactamase-inhibitor | Cefuroxime and Clavulanic acid | J01DC02 | Not Recommended | No |
|  | Amoxicillin and Clavulanic acid | J01CR02 | Not recommended | Yes |
| Imidazoles | Metronidazole | P01AB01 | Access | Yes |
| First-generation-cephalosporins | Cefradine | J01DB09 | Access | Yes |
|  | Cefadroxil | J01DB05 | Access | No |
| Lincosamides | Clindamycin | J01FF01 | Access | Yes |
| Oxazolidinones | Linezolid | J01XX08 | Reserve | Yes |
| Nitrofuran-derivatives | Nitrofurantoin | J01XE01 | Access | Yes |
| Rifamycins | Rifaximin | A07AA11 | Watch | No |
| Fourth-generation-cephalosporins | Cefepime | J01DE01 | Watch | No |
| Carbapenems | Meropenem | J01DH02 | Watch | Yes |
| Tropical antibiotic | Mupirocin | D06AX09 | Access | Yes |

**Supplementary table 4C: Categorized reported health-symptoms under each symptom group**

| **Symptom groups** |  | **Symptoms included** |
| --- | --- | --- |
| Upper respiratory tract infection |  | Cold cough, runny nose, sore throat, nasal congestion, fever, sneezing, tonsillitis, difficulty in swallowing, stuffy nose, sinus infections, infection in the ear |
| Fever |  | Only fever |
| Uncomplicated external skin and soft tissue infections |  | Infections in the wound site, boils, abscesses, infections in skin, redness and swelling with discharge |
| Gastrointestinal infections |  | Diarrhea, abdominal cramping accompanied by loose stools, dysentery and nausea |
| Urinary tract infection |  | Pain and burning during urination, lower abdominal pain with small amounts of frequent urination and burning sensation |
| Lower respiratory tract infections |  | Bronchitis, pneumonia, bronchiolitis |
| Enteric fever |  | Typhoid fever |
| Eye infection |  | Conjunctivitis, eyelashes sticking together due to discharge, swelling with discharge |
| Infections in the oral cavity |  | Ache in the oral cavity, gum swelling with pus, swelling or pus around a tooth |
| Others |  | Piles, ulcers, cardiovascular infections, pimples |
